# Refractory ventricular arrhythmias during aortic valve replacement and cardiac artery bypass requiring 16 attempts of electrical cardioversion: a case report

**DOI:** 10.1186/s40981-020-00369-w

**Published:** 2020-08-11

**Authors:** Mitsuharu Kodaka, Tetsu Mori, Junko Ichikawa, Kazuyoshi Ando, Makiko Komori

**Affiliations:** Department of Anesthesiology & Intensive Care, Tokyo Women’s Medical University Medical Center East, Arakawa-ku, Tokyo, 116-8567 Japan

**Keywords:** Refractory ventricular fibrillation, Nifekalant, Amiodarone

## Abstract

**Background:**

We report a patient in whom we failed to suppress ventricular fibrillation (VF) using nifekalant but succeeded using amiodarone during cardiopulmonary bypass (CPB).

**Case presentation:**

A 65-year-old male with hemodialysis complained of dyspnea and was diagnosed with aortic valve stenosis and angina pectoris; he was opted for elective aortic valve replacement. When the aortic forceps were declamped during CPB, immediate VF was observed; several attempts of electrical cardioversion (EC) with lidocaine and landiolol and three administrations of nifekalant were temporarily effective. However, the rhythm subsequently changed to torsades de pointes. We administered 2 g of magnesium sulfate followed by three doses of amiodarone and initiated continuous infusion. Furthermore, we initiated the pacemaker and intra-aortic balloon pumping. These procedures seemed to be effective; the sinus rhythm was sustained until the end of the surgery.

**Conclusion:**

We experienced a cardiac surgery requiring 16 EC attempts to terminate the life-threatening arrhythmias using amiodarone.

## Background

Nifekalant, a K^+^ channel blocker available only in Japan, is often used in place of amiodarone for suppressing the signs of life-threatening arrhythmias [1] such as ventricular fibrillation and tachycardia (VF and VT). In our department, nifekalant is the first-choice drug in such situations. It has no inotropic effect and has a short half-life (1.5–2 h). Amiodarone has been widely used worldwide for treating such lethal arrhythmias through advanced cardiovascular life support (ACLS). There is limited research comparing the use of both these antiarrhythmic agents for resuscitation [2], and the obtained results have not yet confirmed which drug is more excellent for recovery of spontaneous circulation (ROSC) [3]. Herein, we report a case of a patient where we failed to suppress VF with nifekalant but succeeded with amiodarone, and the patient finally required 16 attempts of electrical cardioversion (EC) and intra-aortic balloon pumping (IABP) after cardiopulmonary bypass (CPB) in cardiac surgery.

## Case description

A 65-year-old male who complained of dyspnea was diagnosed with hypertrophy based on X-ray findings and was hospitalized emergently for an acute heart failure. His past medical history included hypertension and 8 years of hemodialysis treatment due to chronic kidney disease (stage 5GD) by unknown causes. His height and weight were 176 cm and 55 kg, respectively. His laboratory data were abnormal as follows: C-reactive protein, 1.84 mg/dl; N-terminal pro-brain natriuretic peptide, > 35,000 pg/ml; troponin T, 0.111 ng/ml; BUN, 58.2 mg/dl; and Cr, 9.2 mg/dl. Cardiothoracic ratio was 63% based on chest X-ray with butterfly shadow. Electrocardiogram indicated a sinus rhythm, a heart rate of 101 bpm, and a QS pattern in V2–3. Transthoracic echo showed the following findings: ejection fraction (EF), 59%; left ventricular internal dimension in diastole/systole (LVDd/Ds), 57/30 mm; interventricular septum thickness, 20 mm; posterior LV wall thickness, 15 mm; diameter of left atrium, 56 mm; LV mass index, 201 g/m^2^; aortic valve pressure gradient, 104/54 mmHg (max/mean); aortic valve area, 0.54 cm^2^; and aortic regurgitation mild, mitral regurgitation mild, and tricuspid regurgitation mild (pressure gradient, 27 mmHg). The patient’s coronary angiography result indicated stenosis of left anterior descending branch #6, 75%, and left circumflex coronary artery #11, 75%, and computed tomography revealed left-sided pleural effusion. Finally, he was diagnosed with aortic valve stenosis (AS) and angina pectoris, and it was decided to perform elective surgery of aortic valve replacement (AVR) and cardiac artery bypass.

**Fig. 1:**
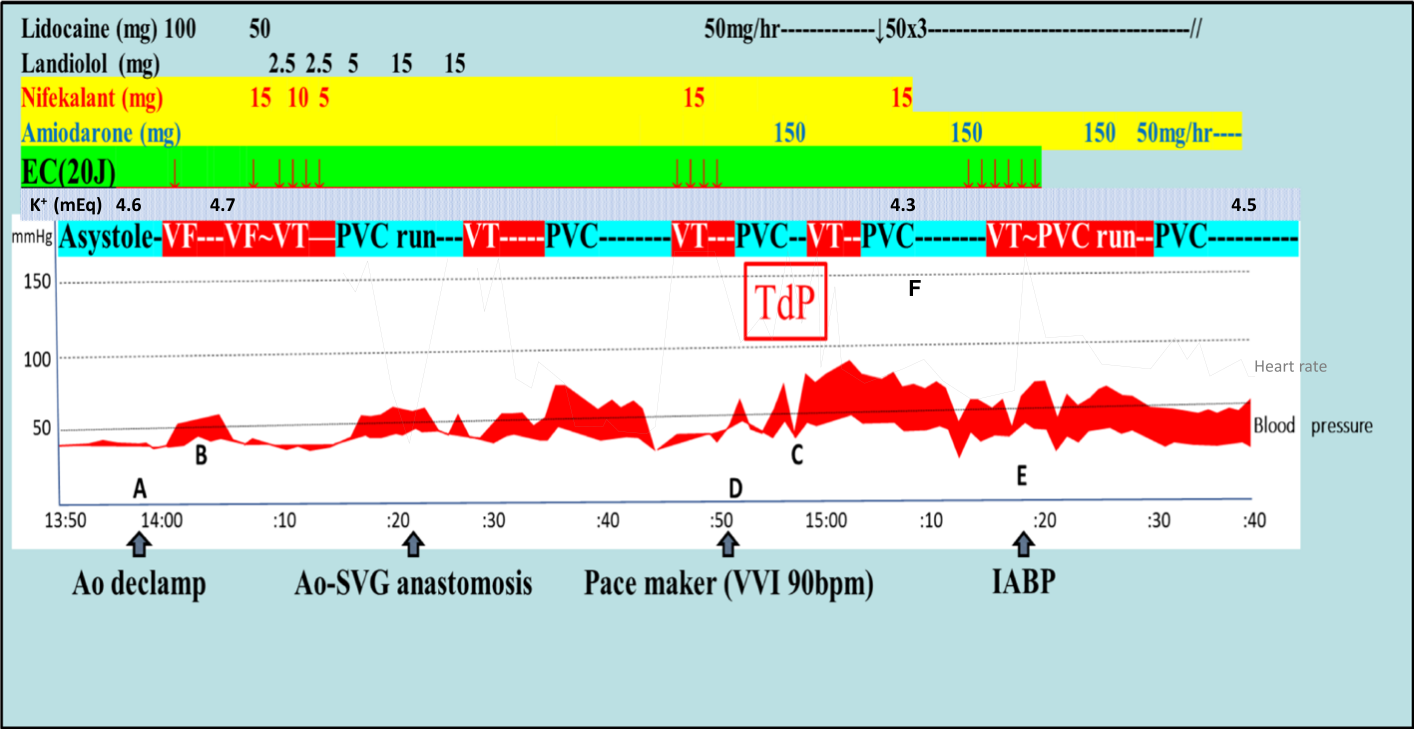
Treatment details for refractory ventricular arrhythmias occurred after aortic declamp. EC, electrical cardioversion; VF, ventricular fibrillation; VT, ventricular tachycardia; PVC, premature ventricular contraction; PVC run, three or more consecutive PVCs; TdP, torsades de pointes; Ao, aorta; SVG, saphenous vein graft; IABP, intra-aortic balloon pumping

After the initiation of CPB and aortic clamping, cardiac arrest was induced immediately after cardioplegia infusion and maintained with 500–700 ml infusion every 30 min. Left internal thoracic artery–left anterior descending branch (LITA–LAD) and saphenous vein–right coronary artery (SVG–RCA) anastomoses and AVR (Magna Ease^TM^ 23 mm) were performed. When the aortic forceps were declamped at 13:57 (marked “A” in Fig. 1), VF occurred immediately as shown in Fig. 1 (anesthetic record). Several attempts of EC with lidocaine (100 and 50 mg) and landiolol (2.5–15 mg) and three times of administrations of nifekalant (5–15 mg) were temporarily effective at 14:12–25 (marked “B”), and the patient ECG suggested sinus rhythm with premature ventricular counts. However, the sinus rhythm and VT occurred alternatively, and the rhythm subsequently changed to torsades de pointes (TdP), which might have occurred because of the overdose of nifekalant at around 14:55 (marked “C”). Therefore, we administered 2 g of magnesium sulfate followed by amiodarone three times, reaching a total of 450 mg (each dose of 150 mg), and initiated continuous infusion at 50 mg/h (c.f. Fig. 1). We also initiated the pacemaker (VVI setting) and IABP at 14:50 and 15:16 (marked “D” and “E”), respectively.

All these procedures appeared to be effective at terminating the VT and subsequently succeeded in weaning from CPB. Thus, the situation with the sinus rhythm was sustained until the end of surgery. During this period, we also took caution to maintain normothermia with no electrolyte imbalance. The flow of the two bypass grafts was assessed using a flowmeter, i.e., LITA–LAD, 50 ml/min, and SVG–RCA, 9 ml/min. Transesophageal echocardiography revealed only diffuse hypokinesis with approximately 20% of EF just after aortic declamping, which gradually improved toward the weaning of CPB to approximately 30% of EF. After admission to the intensive care unit (ICU), the patient showed no VF or VT under the continuous infusion of amiodarone (50 mg/h), and the IABP was withdrawn on postoperative day (POD) 1 and extubated on POD 2. The patient was discharged from the ICU on POD 3 and from the hospital on POD 12 without any complication.

## Discussion

Patients with AS sometimes encounter insufficient cardioplegia because of hypertrophy during CPB. In this case, the patient’s condition was also complicated with coronary artery stenosis and hemodialysis, which sometimes tend to calcify the entire artery. The other causes of lethal arrhythmias are abnormality of electrodes, hypothermia, and air embolism in coronary arteries. The typical treatments for VF include EC and antiarrhythmic drugs such as K^+^ channel blockers. Nifekalant, a potassium channel blocker, is an antiarrhythmic agent approved in Japan since 1999 [4], induced earlier than intravenous amiodarone, which was also acknowledged in 2007. The advantages of nifekalant are that it is a pure potassium channel blocker and therefore does not cause inotropic effect and that it has a shorter half-life than amiodarone, a multichannel blocker. In contrast, the major drawback is the prolongation of QTs by > 0.55 s [5] occasionally due to overdose, which can often induce TdP syndrome as occurred in the present case. Although some studies [2, 3] show that nifekalant is not inferior to amiodarone in managing patients with fatal ventricular arrhythmias, only a few large-scale studies have compared the efficacy of the two drugs. In fact, a couple of similar case reports published in Japan have described that the majority of VF cases had occurred during coronary reperfusion therapy [6] and in patients undergoing bypass surgery [7, 8].

On encountering cases of VF or pulseless VT, nifekalant may be the first choice of treatment for certain anesthesiologists because of its longer history of domestic approval and its basic pharmaceutical advantages, such as faster onset and shorter half-life than amiodarone [2]. Based on their research, Amino et al. [3] reported that it was difficult to conclude which antiarrhythmic agent was more excellent for resuscitation comprising VF or pulseless VT. We inferred that it was difficult to exclude the influences of the pacemaker and IABP for the recovery of the patient. Amiodarone is not only a potassium but also a multichannel blocker and could have been more effective than nifekalant for treating such life-threatening arrhythmia, which had continued for almost 50 min and required 16 attempts of cardioversion during CPB weaning.

A limitation of our anesthetic managements was that we should have reduced the doses of nifekalant to approximately 50–60% while considering hemodialysis [9], even during dilution by CPB. Our study did not measure whether QTc was > 550 ms when changing treatment from nifekalant to amiodarone. Furthermore, after the occurrence of TdP at point “C,” the administration of nifekalant again should have been avoided as indicated by “F” (around 15:10) in Fig. 1 [10].

## Conclusions

We report a patient in whom we failed to suppress VF using nifekalant but succeeded using amiodarone, and the patient finally required 16 attempts of electrical cardioversion and intra-aortic balloon pumping during CPB in cardiac surgery.

## Data Availability

Not applicable

## Abbreviations

EC: Electrical cardioversion
VF: Ventricular fibrillation
VT: Ventricular tachycardia
IABP: Intra-aortic balloon pumping
CPB: Cardiopulmonary bypass
ACLS: Advanced cardiovascular life support
ROSC: Recovery of spontaneous circulation
AS: Aortic stenosis
AVR: Aortic valve replacement
PVC: Premature ventricular contraction
TEE: Transesophageal echocardiography
EF: Ejection fraction
ECG: Electrocardiogram
ICU: Intensive care unit
